# Ulvan-Based Nanofibrous Patches Enhance Wound Healing of Skin Trauma Resulting from Cryosurgical Treatment of Keloids

**DOI:** 10.3390/md20090551

**Published:** 2022-08-26

**Authors:** Stefanos Kikionis, Marianna Koromvoki, Anna Tagka, Eleni Polichronaki, Alexandros Stratigos, Antonios Panagiotopoulos, Aikaterini Kyritsi, Vangelis Karalis, Andreas Vitsos, Michail Rallis, Efstathia Ioannou, Vassilios Roussis

**Affiliations:** 1 Section of Pharmacognosy and Chemistry of Natural Products, Department of Pharmacy, School of Health Sciences, National and Kapodistrian University of Athens, Panepistimiopolis Zografou, 15771 Athens, Greece; 2 Section of Pharmaceutical Technology, Department of Pharmacy, School of Health Sciences, National and Kapodistrian University of Athens, Panepistimiopolis Zografou, 15784 Athens, Greece; 3 First Department of Dermatology-Venereology, National and Kapodistrian University of Athens, Andreas Syggros Hospital, 5 Ionos Dragoumi Str., 11621 Athens, Greece

**Keywords:** ulvan, electrospun nanofibrous patches, keloids, cryosurgery, wound healing

## Abstract

Keloids are skin fibroproliferative disorders, resulting from abnormal healing of deep cutaneous injuries. Cryosurgery, the most common treatment for keloids, causes skin traumas. Even though the clinical practice of cryosurgery has increased, effective wound healing therapy is still lacking. In this investigation, nonwoven nanofibrous patches composed of ulvan, a marine sulfated polysaccharide exhibiting anti-inflammatory and antioxidant activities, and polyethylene oxide (PEO) were fabricated through electrospinning and characterized. Their wound healing efficacy on skin traumas resulting from cryosurgical treatment of keloids was clinically tested and evaluated in comparison to a reference product. Twenty-four volunteer patients undergoing cryosurgery as a treatment of keloids were selected to apply either the ulvan/PEO patch or the reference product for 21 days. The ulvan/PEO patch, 21 days after cryosurgery, showed significant wound healing, elimination of skin inflammation, restoration of biophysical parameters similar to normal values and significant decrease in haemoglobin concentration, skin texture and volume, while no discomfort or adverse reaction was observed. In contrast, the reference product showed inferior performance in all evaluated parameters. The designed ulvan/PEO patch represents the first wound dressing to effectively heal skin trauma after cryosurgical treatment of keloids.

## 1. Introduction

Keloids, from the clinical perspective, appear as elevated, firm, bosselated papules associated with skin color variations, including erythematous or brown pigmentation [1,2]. In contrast to normal and hypertrophic scarring, keloids extend beyond the borders of the original injury [3,4]. A variety of epidermal to dermal insults have been implicated, including surgical incision, burn, trauma, body piercing, insect bite, vaccination and inflammatory acne [1,5,6].

Cryosurgery is an effective and quick treatment of keloids that is performed by topically applying liquid nitrogen in order to freeze the targeted tissue [7]. It induces tissue damage, including damage on blood vessels and capillaries. Further lesion is observed by the formation of ice crystals between cells, osmotic degradation and cellular membrane disruption. However, the most common side effect of cryosurgery is skin trauma [8].

Clinical practice guidelines for the treatment of skin trauma resulting from cryosurgery include the application of a variety of topical agents [8,9], with surveys suggesting that topical products, such as sprays and gels, should be used to promote tissue repair. The main disadvantage of the application of such products is the non-controlled dosage, which frequently leads to incomplete healing [10,11]. In contrast, topical patches, which have not yet been studied for the wound healing of patients’ skin trauma resulting after treatment of keloids with cryosurgery, could offer the advantage of either the constant contact with bioactive wound dressings or the controlled delivery of anti-inflammatory and wound healing agents [12,13,14,15,16,17]. At the same time, such dressings could effectively protect the treated areas from microbial contamination [18,19,20].

Efficient wound treatment could be addressed by the application of innovative, effective wound dressings that can accelerate wound healing with minimum scar marks [21]. Recently, electrospun polymeric nanofibers have emerged as a new technological platform for the development of innovative fibrous matrices for drug delivery, tissue engineering, and wound healing applications [22,23,24]. Tailor-made nanofibrous dressings of various biocompatible and biodegradable, natural or synthetic polymers, can be easily fabricated from electrically charged polymer solutions or melts under the application of a high voltage electric field [25,26,27]. Exhibiting high porosity, high surface area to volume ratio, and structural analogy to the natural extracellular matrix, electrospun nanofibrous nonwovens incorporating various biologically active agents, have emerged as promising candidates for the development of interactive wound dressings able not only to cover the wound, but also to stimulate the wound healing process [19,28,29,30].

Marine polysaccharides are highly biocompatible, biodegradable, non-toxic and abundant biopolymers, with diverse structures and bioactivities, representing valuable biomaterials for the development of novel biomedical systems [31,32,33,34]. Among them, ulvan, the main component of the cell walls of green macroalgae of the genus *Ulva* [35,36], is a complex anionic polysaccharide that has attracted significant interest over the years in the medicinal and pharmaceutical sectors for its utilization in hybrid structures, polyelectrolyte complexes, 2D membranes, and polymeric nanofibers [37,38,39,40,41,42,43,44,45,46,47]. Ulvan, exhibiting antioxidant, antiviral, anti-inflammatory, antihyperlipidemic and anticoagulant activities [47,48,49] and incorporating in its backbone structure rare carbohydrates, such as iduronic acid and sulfated rhamnose [50] that can enhance its wound healing activity, is considered a precious biomaterial for the development of novel wound dressings for the treatment of various skin infections [22,51,52,53].

The aim of the present study was to prepare ulvan-based nanofibrous patches and clinically evaluate their wound healing efficacy, in comparison to a reference product, for the treatment of skin trauma in patients receiving cryosurgery for the removal of keloids.

## 2. Results

The promising results observed in our recent study for the treatment of burn wounds using ulvan/gelatin-based nanofibrous patches [54] prompted us to develop ulvan-based nonwovens and clinically evaluate their wound healing efficacy on skin trauma caused on patients receiving cryosurgery for the removal of keloids.

**Figure 1:**
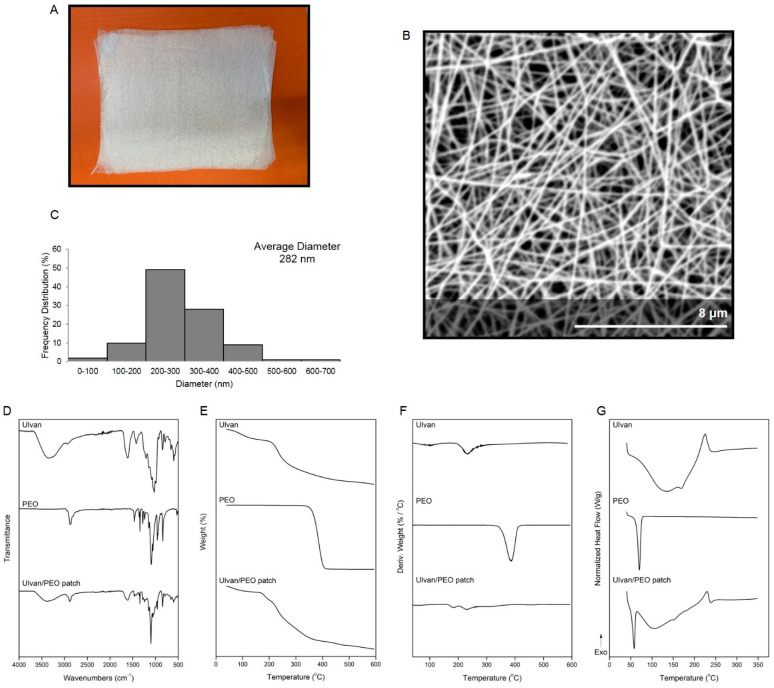
(**A**) Image of the ulvan/PEO nanofibrous patch. (**B**) SEM image of the ulvan/PEO nanofibrous patch at x15,000 magnification. (**C**) Diameter distribution histogram of fibers in ulvan/PEO dressings. (**D**) FT-IR spectra of ulvan, PEO and the ulvan/PEO nanofibrous patch. (**E**) TGA, (**F**) DTG and (**G**) DSC thermograms of ulvan, PEO and the ulvan/PEO nanofibrous patch.

Nonwoven nanofibrous patches were fabricated by electrospinning of a single spinning solution composed of ulvan and polyethylene oxide (PEO) under optimized electrospinning conditions and parameters (Figure 1A). The analysis of the SEM images of the ulvan/PEO patches revealed a uniform beads-free fibrous network with smooth fibers of cylindrical shape morphologies (Figure 1B). The fiber size diameters ranged from 47 to 621 nm, with an average diameter size of 282 ± 86 nm (Figure 1C). The uniform web structure indicated a homogeneous distribution of ulvan within the fibrous matrix, resulting in a highly porous surface area composed of ultrafine fibers that could facilitate the diffusion of ulvan onto the wounded skin, enhancing the wound healing efficacy of the patch [29].

The FTIR spectra of ulvan powder, PEO powder and the fabricated ulvan/PEO patch are shown in Figure 1D. For ulvan, the characteristic absorption bands attributed to the stretching vibrations of −OH, −C=O carboxylic groups and C−O−C ether glycosidic linkage were observed at 3346, 1614 and 1033 cm^−1^, respectively, whereas the sulfate ester groups showed characteristic absorption bands at 1207 and 845 cm^−1^. In the FTIR spectrum of PEO, the characteristic peak attributed to the −CH_2_ stretching vibration was recorded at 2874 cm^−1^, whereas the characteristic peaks attributed to the C–O–C stretching vibrations were observed at 1092, 959 and 842 cm^−1^. The FTIR spectrum of the ulvan/PEO fibers displayed the characteristic bands of its components. The characteristic signals were observed at slightly shifted bands compared to the raw materials, indicating their interaction into the hybrid polymer matrix. The successful incorporation of ulvan in the fiber mats was evident by its characteristic absorption bands at 3375 and 1615 cm^−1^ assigned to the stretching vibrations of −OH and −C=O groups, respectively, while the presence of PEO was verified by the characteristic peak at 2878 cm^−1^ [39,40,45,54,55].

The fabricated ulvan/PEO nanofibrous patches, as well as the utilized ulvan and PEO raw materials, were physicochemically characterized by TGA and DSC analyses (Figure 1E–G). As shown in the TGA thermograms (Figure 1E), ulvan, after a slight mass loss attributed to the volatilization of moisture and hydrogen-bound water, started to decompose at 210 °C, whereas the decomposition of PEO was initiated at 347 °C. The ulvan/PEO fibers showed a different thermal profile due to the synergistic thermal events of their ingredients, with an initial dehydration mass loss, a small degradation step at 171 °C and a major decomposition step at 214 °C. The different thermal behavior of the ulvan/PEO nanofibers was also evident in the derivative thermogravimetry (DTG) thermograms (Figure 1F). The maximum decomposition was recorded at 233 °C for ulvan, 385 °C for PEO, and at 231 °C for the ulvan/PEO fibers. In the DSC thermograms (Figure 1G), ulvan showed the characteristic thermal events of polysaccharides, with a broad endotherm below 131 °C attributed to dehydration phenomena and a degradation exothermic peak at 223 °C, whereas PEO showed a melting endothermic peak at 70 °C. The ulvan/PEO nanofibers showed an endothermic melting band at 58 °C and a broad dehydration endotherm at 103 °C, followed by a broad degradation exotherm at over 230 °C, revealing synergistic degradation events of the combined materials in its thermal profile [30,45,55,56].

Demographic information, including age, body-mass index (BMI), phototype (Fitzpatrick skin type), medical conditions, family history, sun exposure, and smoking, and full medical history, as recorded for all patients, are presented in Table 1.

**Figure 2:**
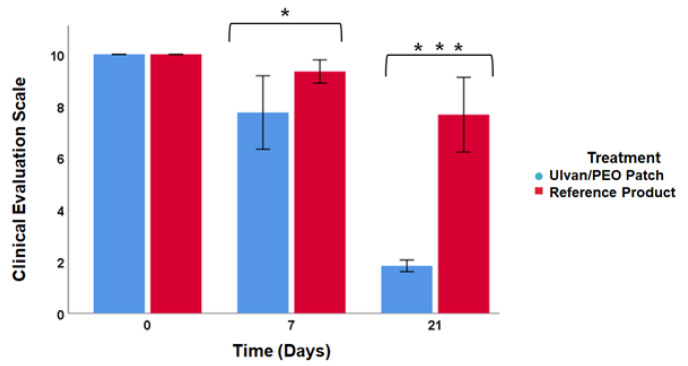
Clinical evaluation of the two treatments based on the POSAS criteria, on days 7 and 21 after cryosurgery. Significant differences were observed between the two interventions throughout the treatment period (* *p* < 0.05, *** *p* ≤ 0.001). Complete skin recovery was observed in the ulvan/PEO patch patients 21 days after cryosurgery.

The clinical assessment of skin trauma using the POSAS scar scale [57,58] is presented in Figure 2. On day 7 after cryosurgery, skin trauma was decreased, color and volume were improved and the POSAS score was 8 for the ulvan/PEO patch patients, while the reference product patients had higher trauma surface, redness, scar volume and a POSAS score of 9. On day 21 after cryosurgery, the POSAS score for the ulvan/PEO patch patients was 2, without signs of skin trauma, while for the reference product patients the POSAS score was 8. At both time points, the POSAS score was significantly different between the two treatments (day 7, *p* = 0.024; day 21, *p* = 0.001).

**Figure 3:**
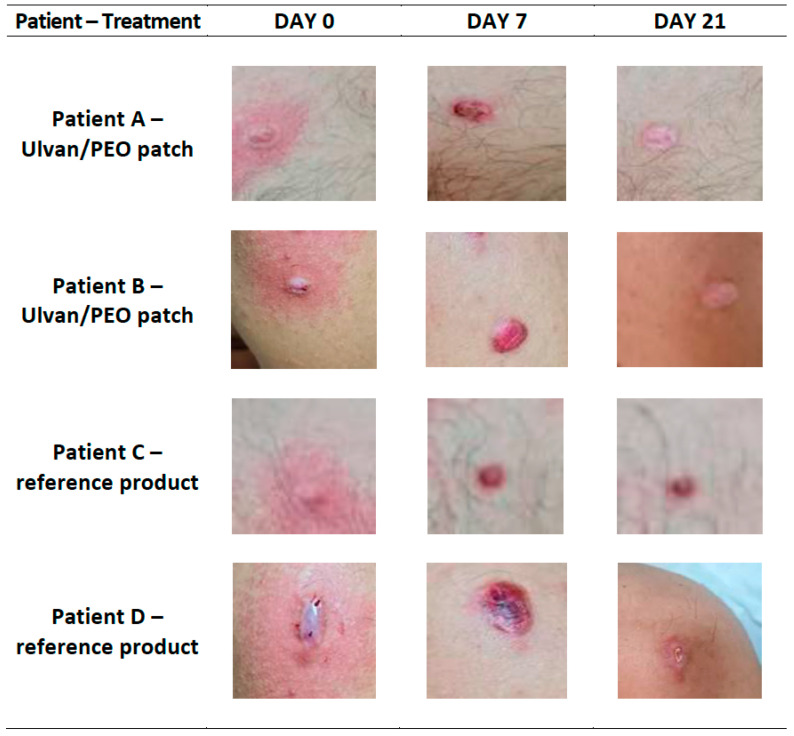
Representative images, at day 0 (receiving cryosurgery), day 7 and day 21, of (**A**) a 19-year-old male patient treating the trauma from the removal of a pectoral keloid with the ulvan/PEO patch, (**B**) a 29-year-old male patient treating the trauma from the removal of a shoulder keloid with the ulvan/PEO patch, (**C**) a 19-year-old male patient treating the trauma from the removal of a pectoral keloid with the reference product, and (**D**) a 29-year-old male patient treating the trauma from the removal of a shoulder keloid with the reference product. In contrast to the reference product, the ulvan/PEO patch demonstrated significant anti-inflammatory efficacy and wound healing.

Photo-documentation confirmed the initial evaluation based on the POSAS criteria. In contrast to the reference product, the ulvan/PEO patch significantly contributed to wound healing and soothed the injured skin sites (Figure 3).

**Figure 4:**
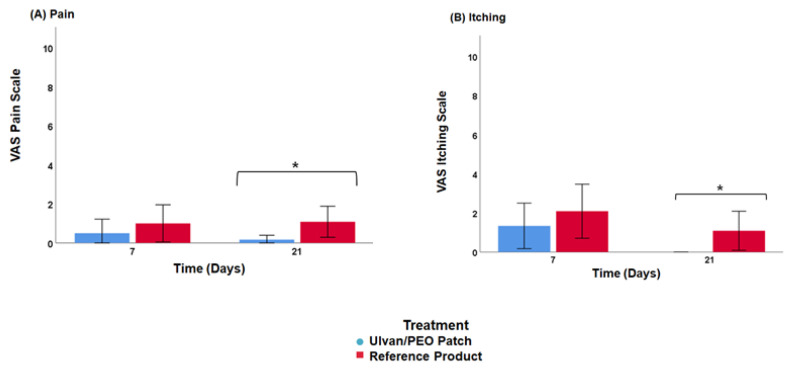
Patient self-assessment of (**A**) pain and (**B**) itching using a visual analog scale (VAS) on day 7 and 21 after cryosurgery (* *p* < 0.05).

Significant differences between the two treatments were observed on day 21 (* *p* < 0.05). In contrast to patients treated with the reference product, almost no pain and no itching was reported from patients using the ulvan/PEO patch (Figure 4).

**Figure 5:**
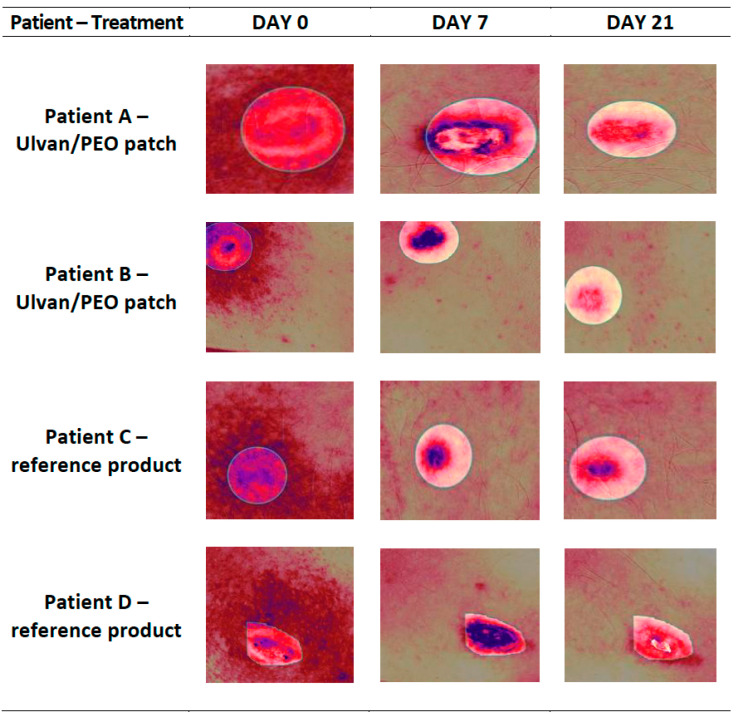
Representative Antera 3D camera images, at day 0 (receiving cryosurgery), day 7 and day 21, of (**A**) a 19-year-old male patient treating the trauma from the removal of a pectoral keloid with the ulvan/PEO patch, (**B**) a 29-year-old male patient treating the trauma from the removal of a shoulder keloid with the ulvan/PEO patch, (**C**) a 19-year-old male patient treating the trauma from the removal of a pectoral keloid with the reference product, and (**D**) a 29-year-old male patient treating the trauma from the removal of a shoulder keloid with the reference product. In contrast to the reference product, the ulvan/PEO patch showed significant anti-inflammatory effect.

According to the images analyzed with the Antera 3D software (Figure 5), the ulvan/PEO patch exerted a remarkable anti-inflammatory activity after cryosurgery, contributing to the wound healing process.

**Figure 6:**
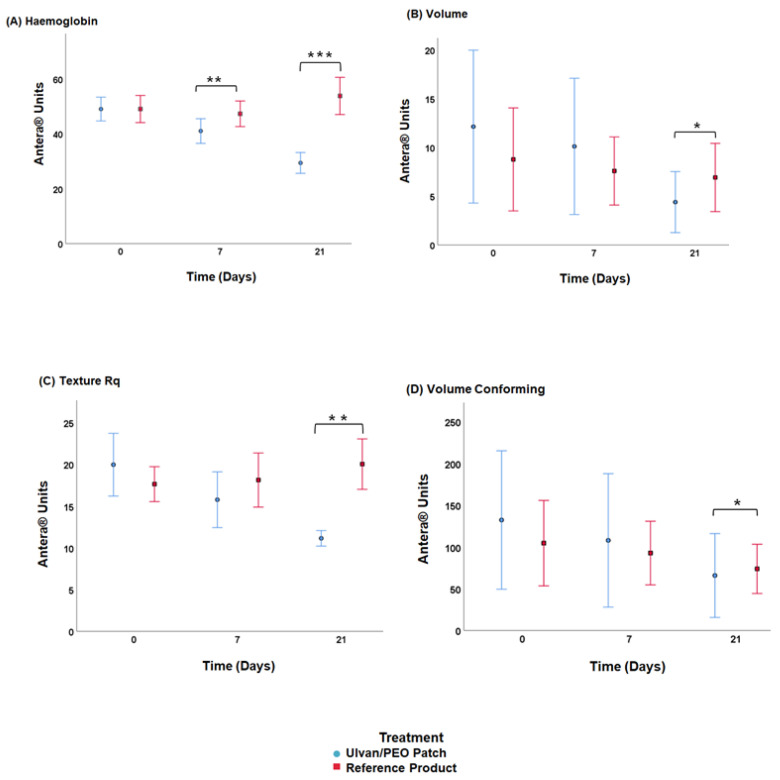
(**A**) Haemoglobin concentration, (**B**) volume, (**C**) skin texture, and (**D**) volume conforming at day 0 (receiving cryosurgery), day 7 and day 21 (* *p* < 0.05, ** *p* ≤ 0.01, *** *p* ≤ 0.001).

The patients treated with the ulvan/PEO patch, as compared to those using the reference product, showed lower haemoglobin concentrations that returned close-to-normal values on day 21 (Figure 6A, day 7, *p* = 0.01; day 21, *p* = 0.001). On day 21, volume, volume conforming and skin texture were significantly decreased in the patients using the ulvan/PEO patch in comparison to those using the reference product (Figure 6B–D, *p* = 0.032, *p* = 0.04 and *p* = 0.005, respectively).

**Figure 7:**
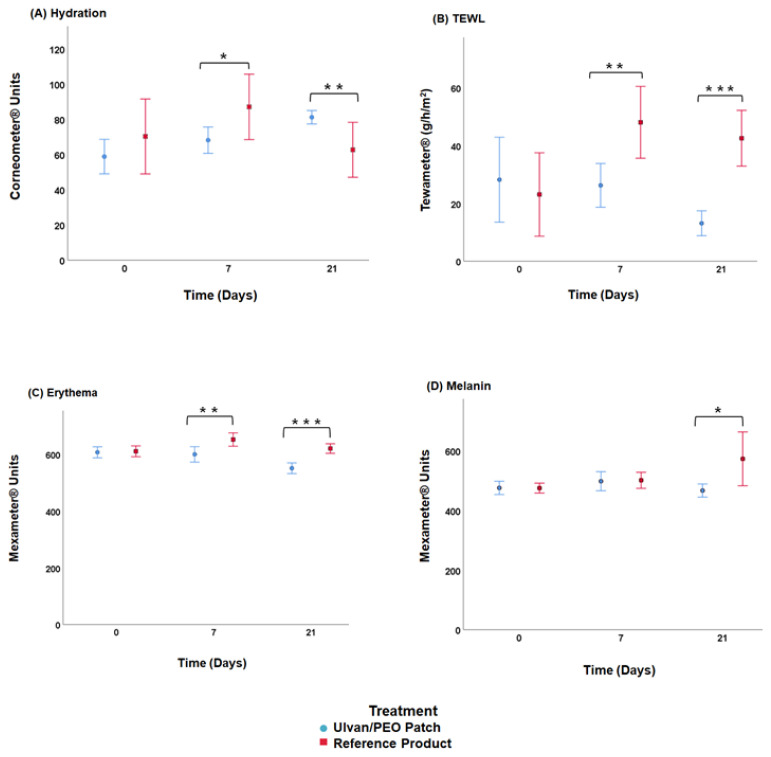
(**A**) Hydration, (**B**) TEWL, (**C**) erythema, and (**D**) melanin, at day 0 (receiving cryosurgery), day 7 and day 21 (* *p* < 0.05, ** *p* ≤ 0.01, *** *p* ≤ 0.001).

The hydration, transepidermal water loss (TEWL), and erythema levels on days 7 and 21, as well as melanin levels on day 21 were significantly lower for the patients using the ulvan/PEO patch, as compared to those using the reference product (Figure 7, *p* < 0.05).

**Figure 8:**
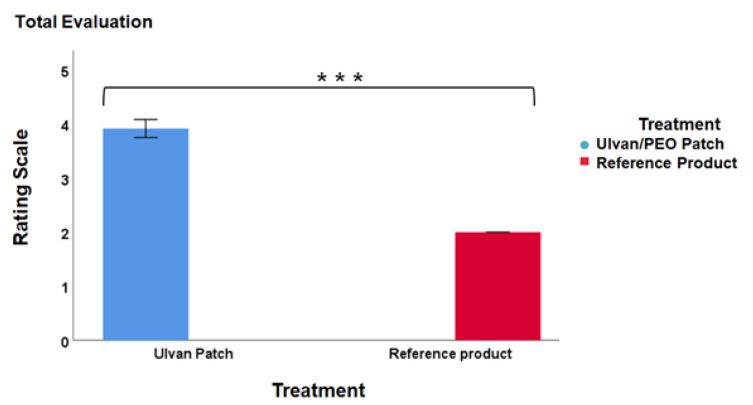
Patient evaluation of the two treatments (ulvan/PEO patch and reference product) based on their main characteristics (color, texture, applicability, ease of use, no irritation, and overall rating). Significant differences were found between the two interventions (*** *p* ≤ 0.001), with patients preferring the ulvan/PEO patch over the reference product.

All patients filled out a questionnaire in which they were asked to comment on their experience using either the ulvan/PEO patch or the reference product. In the overall evaluation, patients using the ulvan/PEO patch reported significantly higher satisfaction (4/5) in comparison to those using the reference product (Figure 8, *p* = 0.001).

## 3. Discussion

In the present study, ulvan-based nanofibrous patches were fabricated, characterized (Figure 1) and evaluated for their ability to efficiently heal wounds in patients undergoing cryosurgery for the removal of keloids.

According to the characteristics of the patients participating in the study (Table 1), including demographics and characteristics of the disorder, no significant differences were observed between the patients using the ulvan/PEO patch and those using the reference product. It is of interest to note that keloids were almost exclusively (87.5%) developed on acne patients. Other studies have also reported that acne is an important factor in the development of keloids [6].

All patients involved in this study presented improvement during the clinical evaluation (Figure 2). However, in the case of patients using the ulvan/PEO patch, all POSAS parameters were significantly decreased, achieving a value of 2 by day 21, which is very close to that of normal skin condition (value of 1). In contrast, for patients treated with the reference product, the respective POSAS value was much higher, closer to that of worst scar. Concerning the important wound parameters of pain and itching, the ulvan/PEO patch treatment showed a total elimination of itching and a significant decrease in pain (Figure 4). According to our findings, the healing activity exerted by the ulvan/PEO patch was independent to the anatomical site (Figure 3).

Photo-documentation with an Antera 3D camera showed that the ulvan/PEO patch, in comparison to the reference product, resulted in a significant decrease in wound haemoglobin levels, indicating a remarkable anti-inflammatory activity (Figure 5 and Figure 6A) [19,30]. In parallel, skin texture, volume and volume conforming significantly decreased when the ulvan/PEO patch was used (Figure 6B–D), highlighting the progress in the wound healing process. The use of the Antera 3D camera, employed for the first time in this study in order to assess wound healing in patients after cryosurgery, showed a noticeable sensitivity in the estimation of the above parameters.

The biophysical parameters of the skin, namely, wound skin barrier function, stratum corneum hydration, erythema and melanin, were almost re-established to normal levels when patients received treatment with the ulvan/PEO patch (Figure 7). It has been reported that hydration, TEWL and erythema, and to a lesser extent melanin, are directly related to the severity of inflammation [6]. The obtained results corroborate the remarkable anti-inflammatory activity of the ulvan/PEO patch.

It is noteworthy to mention that the ulvan/PEO patch was highly appreciated by the patients (Figure 8). No irritation was reported by the patients and no adverse reaction was observed either by clinical evaluation or by the patients, supporting the safe utilization of the ulvan/PEO patch as a wound dressing.

In summary, the application of the designed ulvan/PEO patch for 21 days on skin trauma resulting from the cryosurgical treatment of keloids led to remarkable wound healing with concomitant elimination of the skin inflammation. The significant decrease in the POSAS score and the restoration of the various parameters related the skin’s condition (hydration, TEWL, erythema, melanin, haemoglobin concentration, skin texture, volume, volume conforming) strongly supported these findings. In addition, no adverse reaction or patient discomfort was observed. In contrast, treatment with the reference product showed inferior performance in all evaluated parameters.

Even though the main limitation of this clinical study is the relatively small sample size, it is obvious that the ulvan/PEO nanofibrous patch, due to the antioxidant, anti-inflammatory and wound healing properties of ulvan [35,49,59,60], greatly contributed to the efficient healing of the skin trauma resulting from the cryosurgery of keloids. Given the promising results of this preliminary study, a clinical trial of the application of the designed ulvan/PEO patch on a larger number of patients receiving cryosurgery as a treatment to keloids or even other skin disorders will be conducted.

## 4. Materials and Methods

### 4.1. Reagents and Raw Materials

Polyethylene oxide (PEO) (MW 8,000,000) was purchased from Sigma-Aldrich (Darmstadt, Germany). Specimens of the green alga *Ulva rigida* were collected in Parga bay, Greece. The algal specimens were cleaned from foreign materials, washed with seawater and fresh water, and air-dried. For the isolation of ulvan, 0.6 kg of the air-dried algal biomass, grounded to small pieces, was soaked in 12 L of distilled H_2_O and heated in an autoclave at 121 °C for 20 min. The resulting hot aqueous solution was filtrated through cotton cloth; the filtrate was left to cool at room temperature and 48 L of ethanol (96% *v/v*) was added to the filtrate for the precipitation of the polysaccharide. Subsequently, the suspension was left at 4 °C overnight and the resulting precipitate was filtered through cotton cloth, washed thoroughly with ethanol, sonicated in an ultrasonic bath for 1 h, vacuum-filtered, and finally lyophilized overnight to afford ulvan as an off-white powder. The characterization of ulvan (MW distribution centered at approx. 1150 kDa; 47.5% sulfate, 40.4% carbohydrates; among carbohydrates rhamnose and uronic acids represented 25.9% and 17.9%, respectively) was performed as previously described [50]. Sterile non-woven gauze was purchased from a local pharmacy store.

### 4.2. Preparation of the Electrospun Nanofibrous Patches

The ulvan/PEO spinning solution was prepared under stirring for 24 h at room temperature to ensure its homogeneity by dissolving ulvan at a concentration of 4% *w/v* in deionized H_2_O followed by the addition of PEO at 2% *w/v*, resulting in a 2:1 (*w/w*) ratio of ulvan to PEO. Electrospinning was performed using a *γ*-High Voltage Research DC power supply generator (Gamma High Voltage Research, Ormond Beach, FL, USA) with a maximum voltage of 50 kV. The polymer solution was electrospun from 10-mL disposable syringe fitted with 23G stainless-steel blunt needle, with the syringe mounted on a horizontally positioned Harvard PHD 2000 programmable syringe pump (Harvard Apparatus, Holliston, MA, USA). The produced nanofibers were deposited on sterile gauge wrapped on a RC-6000 rotating drum collector at a rotation speed of 500 rpm (NaBond Technologies, Hong Kong). The temperature and relative humidity were 22 ± 2 °C and 60 ± 5%, respectively. The solution feeding rate, applied voltage and tip-to-collector distance were fixed at 1 mL/h, 25 kV and 30 cm, respectively. The prepared nanofibrous patches were sterilized under UV light for 1 h prior to their use.

### 4.3. Characterization of the Nanofibrous Patches

A PhenomWorld desktop scanning electron microscope (SEM) with tungsten filament (10 kV) and charge reduction sample holder (Thermo Fischer Scientific, Waltham, MA, USA) was used for the morphological characterization of the nanofibers of the ulvan/PEO patch. The samples were sputter-coated with gold. Using the embedded image analysis software (Fibermetric/Phenom Pro Suite v.2.3, Eindhoven, The Netherlands), the diameters of 100 fibers from each SEM image were measured and the average fiber diameter was determined. The chemical composition of the fibers was analyzed by Fourier transform infrared spectroscopy using the attenuated total reflection (ATR) method on a FTIR Bruker Alpha II (Billerica, MA, USA). Thermogravimetric (TGA) analysis was performed using a TA Thermogravimetric Analyzer (TGA 55, TA instruments, New Castle, DE, USA) at a 10 °C/min heating rate from 40 to 600 °C under a 25 mL/min nitrogen flow. The sample weight, sample temperature, and heat flow were recorded continuously. Differential scanning calorimetry (DSC) analysis was conducted using a TA Thermal Analyzer (Discovery DSC 25, TA instruments, New Castle, DE, USA). Samples of 6–7 mg sealed in aluminum pans were heated from 40 to 350 °C at a constant rate of 10 °C/min under a 50 mL/min nitrogen flow.

### 4.4. Study Design and Patient Selection

All procedures performed were in accordance with the guidelines of good clinical practice (GCP) as defined in Directive 2001/20/EC, the US Federal Code of Users (21 CFR Part 312), and the International Conference on Harmonization (ICH). The study was conducted in accordance with the principles of the Declaration of Helsinki (Directive 2001/83/EC; ICH Issue E9 1996; Directive 2001/20/EC; Directive 2002/98/EC; Directive 2003/63/EC; ICH E (6) R1; 21 CFR Part 312; WHO 2008). The volunteers were selected among the patients of the First Department of Dermatology-Venereology, National and Kapodistrian University of Athens, Andreas Syggros Hospital, from October 2021 to January 2022. The protocol of the study was approved by the Institutional Scientific Review Board of the University Hospital “Andreas Syggros”, National and Kapodistrian University of Athens, Medical School (Protocol. Nr. 3693/14-10-2021). Eligible patients were adults diagnosed with confirmed keloids before receiving cryosurgery therapy. The lesions were located on the shoulders, chest, forearm, back or arms. The exclusion criteria were pregnancy, breastfeeding, concomitant chemotherapy, immunosuppressive treatment, previous radiotherapy to the treated area, as well as patients with other autoimmune skin diseases, including atopic dermatitis, psoriasis, and ichthyosis. This was an open-label, two-treatment clinical research trial comparing the efficacy of the ulvan/PEO patch versus a reference product. Twelve patients were randomly selected to apply the ulvan/PEO patch and twelve patients were randomly selected to apply the reference product in different cryosurgery treated areas.

### 4.5. Cryosurgery and Application of Treatment

The patients received two cycles of local conventional cryosurgery (liquid nitrogen at −196 °C) between 20–30 s. They were instructed to apply for 21 days either the ulvan/PEO patch daily for 24 h or a thin layer of the reference product (topical dermal solution spray with antibacterial, antiseptic and wound healing properties; its principal active agent was octenidine hydrochloride 0.1% *w/w*) twice daily to the skin trauma at different sites [61].

### 4.6. Clinical Assessment

The clinical evaluation included index rating using the Patient and Observer Scar Assessment Scale (POSAS). The scale ranges from 1 (normal skin) to 10 (worst scar imaginable) and assesses scar color, elasticity, hypopigmentation, volume, pain, itching, overall opinion and skin type of the patient [57,58]. All enrolled patients completed the study. Their mean age was 26 years, 29.2% were men, and 79.2% were women. Among the patients, 100% had keloids. Of all patients, 83.3% of those using the ulvan/PEO patch and 91.7% of those using the reference product had developed acne before keloids. A complete medical history and demographic data, including age, body-mass index (BMI), phototype (Fitzpatrick skin type), medical conditions, family history, sun exposure and smoking, were recorded for all patients at acceptance to take part in the study. The most important diagnostic criteria for successful wound healing included the results of clinical assessment of keloids and photo-documentation. Skin inflammation was recorded on day 0 of cryosurgery. Skin images were captured with a Nikon D5100 digital camera (Nikon, Tokyo, Japan) equipped with an AF-S Micro Nikkor 60 mm f/2.8 G ED lens (Nikon, Tokyo, Japan) located at a distance of 33 cm perpendicular to the skin. Associated symptoms, such as pain and itching, were reported by patients on days 0, 7, and 21 thereafter using a visual analogue scale (VAS, 10 cm long; 0 = no symptoms, 10 = very severe symptoms).

### 4.7. Skin Surface Analysis

The skin was examined with an Antera 3D camera (Miravex, Dublin, Ireland) to the symmetric healthy anatomical sites to keloids (day 0) and on days 0, 7, and 21 after cryosurgery to the keloids’ anatomical sites. Haemoglobin concentration, skin texture, and skin volume were assessed using Antera 3D software (Miravex, Dublin, Ireland).

### 4.8. Evaluation of Biophysical Parameters of the Skin

Skin parameters, including hydration, TEWL, erythema and melanin, were assessed by non-invasive biophysical methods to the symmetric healthy anatomical sites to keloids (day 0) and on days 0, 7, and 21, and after cryosurgery to the keloids’ anatomical sites. Hydration was measured by changes in dielectric constant using a corneometer CM 820 (Courage + Khazaka electronic GmbH, Cologne, Germany). Data were recorded in arbitrary units. The skin barrier function (TEWL) was assessed with a Tewameter TM 210 (Courage + Khazaka electronic GmbH, Cologne, Germany) by measuring the density gradient of water evaporation from the skin. The estimation was based on the mean value of the flux density of water (in g/m^2^/h) obtained 1 min after the start of the measurement. Erythema and melanin were calculated using a Mexameter MX 18 (Courage + Khazaka electronic GmbH, Cologne, Germany) by measuring absorption/reflection at three different light wavelengths. The data were recorded in arbitrary units. Before each measurement, the treated area was cleaned with 0.9% sodium chloride solution and wiped with sterile gauze.

### 4.9. Patient Self-Report

The two therapeutic interventions were evaluated by the patients 21 days after cryosurgery using a questionnaire. The overall evaluation of the ulvan/PEO patch and the reference product was assessed using a standardized scale from 0 (maximum negative effect) to 5 (maximum positive effect).

### 4.10. Statistical Analysis

The statistical analysis began with the estimation of descriptive criteria. Before statistical comparisons were made between two or more groups, a normality test was performed. To determine the normality of the data distribution, the Shapiro–Wilk test was used because of the small number of participants. If the nominal normality hypothesis is rejected, the data are considered to deviate from the normal distribution. Because the data in this study were not normally distributed in all situations, non-parametric approaches were used. The Mann–Whitney U test was used to compare two independent groups, while the Wilcoxon signed-rank test was used for group comparison between two time points. Values at subsequent time points were compared using the Friedman non-parametric test. Once a significant result was obtained, a post hoc analysis using Dunn’s test was performed. In this study, the type I error (significance threshold) was set at 5% in all situations. If the estimated p-value (*p*) was less than the significance level, the result was considered significant, p-values of 0.05 were considered statistically significant and are indicated by the symbols (*) for *p* ≤ 0.05, (**) for *p* ≤ 0.01, (***) for *p* ≤ 0.001, and (****) for *p* < 0.0001. SPSS^®^ (v.26, IBM, Chicago, IL, USA) was used for all statistical analyses.

## 5. Conclusions

Cryosurgery is an effective and quick intervention for treating keloids, skin fibroproliferative disorders that result from abnormal healing of deep cutaneous injuries. Skin trauma, often encountered as a side effect of cryosurgery, is, to date, treated with topical products, such as sprays and gels, to promote tissue repair. The main drawback of the application of such products is the uncontrolled dosage, which is frequently associated with incomplete or delayed healing and even infections. This clinical study aimed to present the evaluation of a new ulvan-based nanofibrous wound dressing as an effective treatment of wounds resulting from cryosurgical removal of keloids. Treatment of skin trauma resulting from cryosurgical removal of keloids with the designed ulvan/PEO nanofibrous patch effected remarkable wound healing with concomitant elimination of skin inflammation. These findings were supported by the significant decrease in the POSAS score and the restoration of various parameters related to the skin’s condition, coupled with the lack of adverse reaction or patient discomfort. In contrast, treatment with the reference product showed inferior performance in all evaluated parameters.

## Figures and Tables

**Table 1:** Characteristics of the patients at baseline.

Characteristics of Patients	Ulvan/PEO Patch (*N* = 12)	Reference Product (*N* = 12)	Total Patients (*N* = 24)
**Demographics**			
Median age (interquartile range) (year)	26 (18–46)	26 (18–46)	26 (18–46)
Gender (%)			
Male	25.0	33.3	29.2
Female	75.0	66.6	70.8
Body-mass index (%)			
>25 (overweight)	16.6	25.0	20.8
<25 (healthy weight)	83.3	75.0	79.2
Fitzpatrick skin type (%)			
I	8.3	0.0	4.2
II	66.7	75.0	70.8
III	16.7	16.7	16.7
IV	8.3	8.3	8.3
Keloids and acne (%)	83.3	91.7	87.5
**Characteristics of Disorder**			
Keloid size (%)			
>3 cm^2^	8.3	0.0	4.2
<3 cm^2^	91.6	100.0	95.8
Primary (%)	66.6	66.6	66.6
Recurring (%)	33.3	33.3	33.3

## Data Availability

The data that support the findings of this study are available from the corresponding authors upon request.
